# Supplementary material for: mTOR Driven Gene Transcription Is Required for Cholesterol Production in Neurons of the Developing Cerebral Cortex
Source: Int J Mol Sci. 2021 Jun 3;22(11):6034. doi: 10.3390/ijms22116034 (PMC8199781; doi:10.3390/ijms22116034)
Supplement: Supplementary file 1 [file ijms-22-06034-s001.zip › ijms-1212088-supplementary.pdf]

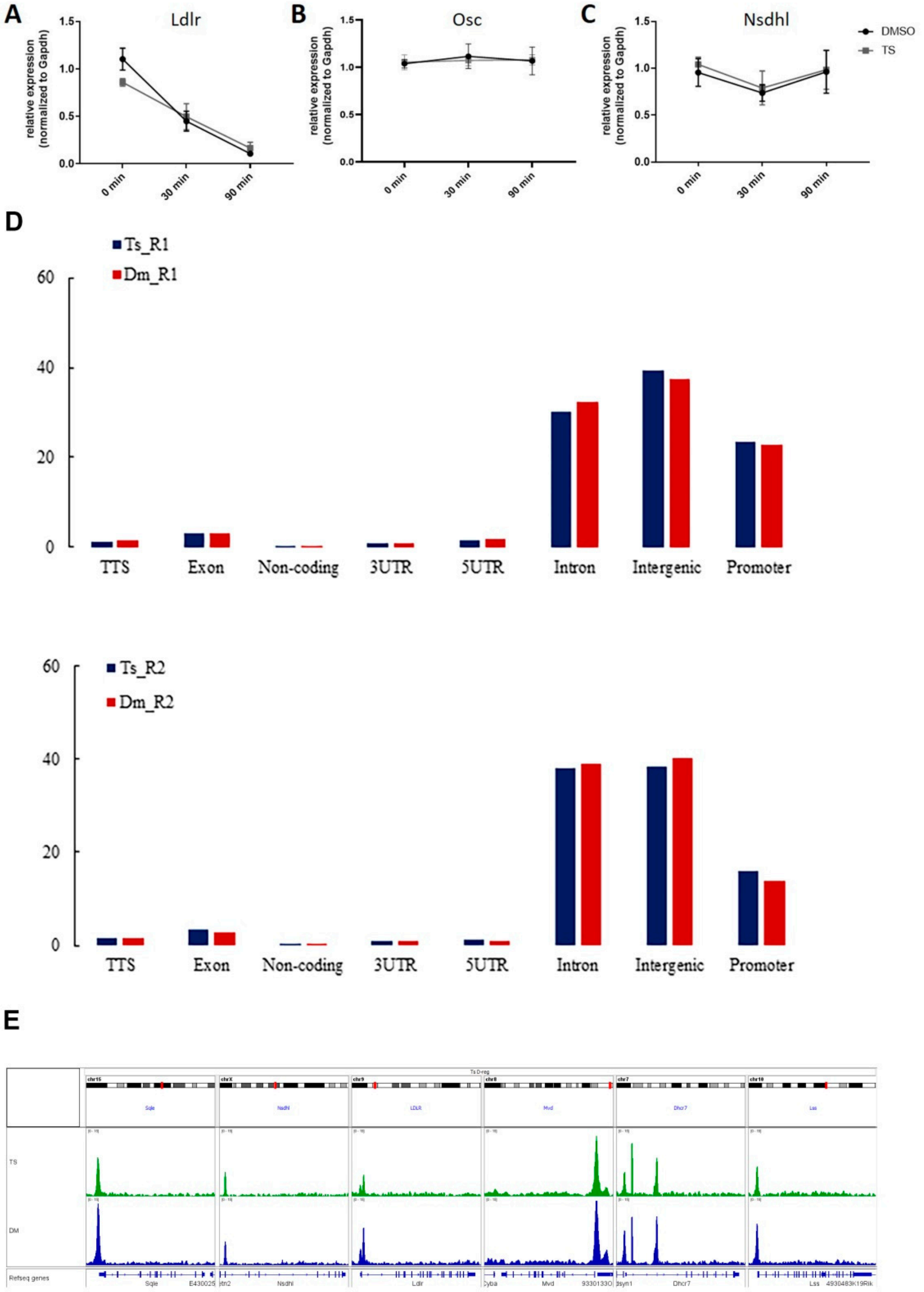

Fig. S1: Temsirolimus treatment does not influence RNA stability or chromatin accessibility in neurons. (A-C) Primary cortical neurons were treated with DMSO or temsirolimus (10 µM) in combination with 5 µg/ml actinomycin D for the indicated times. After cell lysis total RNA was extracted and subjected to RT-qPCR experiments to detect the abundance of Ldlr, Osc and Nsdhl; mRNA expression was normalized to Gapdh. Data represent the average of three biological replicates. (D-E) ATAC-Seq experiments. Primary cortical neurons were treated with DMSO or Temsirolimus (10 µM) for 5 hours followed by cell lysis and ATAC-Seq. (D) Peak distribution of the

two biological replicates of the ATAC-Seq experiments. (E) Representative pictures of ATAC-Seq peaks in the promoters of cholesterol biosynthesis genes.

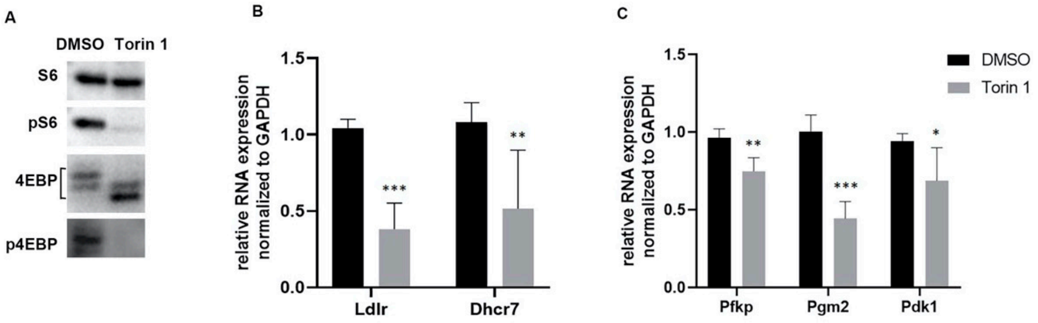

Fig. S2: Torin 1 treatment downregulates expression of cholesterol and glycolysis genes in primary neurons. Primary cortical neurons at DIV6 were treated with Torin 1 (500 nM) or DMSO for 5 hours. (A) Proteins were extracted and westernblot experiments were performed with antibodies detecting S6, pS6, 4EBP and p4EBP. (B, C) Total RNA was extracted and RT-qPCR experiments were performed to detect the abundance of the cholesterol biosynthesis genes Ldlr and Dhcr7 (B) and the glycolysis genes Pfkp, Pgm2 and Pdk1 (C); mRNA expression was normalized to Gapdh. Data represent the average of three biological replicates. For statistical analyses, student's t test was used. *P < 0.05; **P < 0.01; ***P < 0.001.

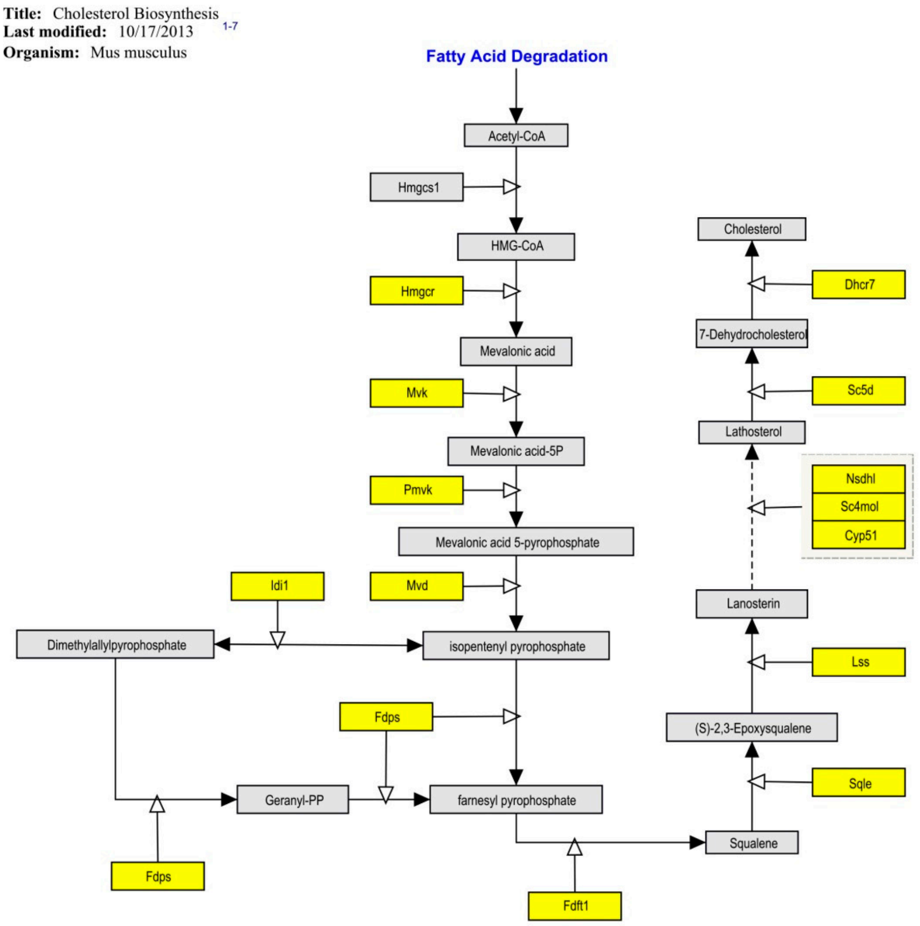

Fig. S3: Temsirolimus treatment reduces cholesterol levels in primary neurons. Schematic overview of the cholesterol synthesis pathway in the mouse. Genes identified as being downregulated in the RNA-Seq experiment after temsirolimus treatment for 24 hours are marked in yellow.

Primer	Sequence (5’-3’)
Sqle_F	catgagtctccggaaagcag
Sqle_R	tgaagcacaacaccttctataaactt
Dhcr7_F	ccagcagaatggcttgttct
Dhcr7_R	aatagatggtgggctccaag
Dhcr24_F	ggtcatgacggacgacgta
Dhcr24_R	agggcttgtagtaactgccaat
Nsdhl_F	cgccatgaagcctattgacta
Nsdhl_R	ttagggtcgttggcatcc
Lss_F	ggggcatacctaccaagatg

Lss_R	ccgctgttcacatgattcaa
Acss2_F	gcctgcaatcctgaatgagt
Acss2_R	cagggctgcttgaacacc
Ldlr_F	agaggggtgaactggtgtga
Ldlr_R	caggtactggcaaccaccat
Mvd_F	ctgaatggtcgcgaggag
Mvd_R	gagtgtccccgtcctctgt
Pfkp_F	gagggaccccatctgcat
Pfkp_R	gtagcttccagcaaggcaat
Pgm2_F	gcgactggaccaggagac
Pgm2_R	gctgtttcactgactcagaggtta
Pdk1_F	gttgaaacgtcccgtgct
Pdk1_R	gcgtgatatgggcaatcc
Gapdh_F	gggttcctataaatacggactgc
Gapdh_R	tacggccaaatccgttcaca

Table S1: Primer sequences
